# Editorial: Recent advances in the treatment of parathyroid disease

**DOI:** 10.3389/fendo.2024.1444651

**Published:** 2024-06-21

**Authors:** Takahisa Hiramitsu, Zia Moinuddin, Titus Augustine

**Affiliations:** ^1^ Department of Transplant and Endocrine Surgery, Japanese Red Cross Aichi Medical Center Nagoya Daini Hospital, Nagoya, Japan; ^2^ Department of Transplant and Endocrine Surgery, Manchester Royal Infirmary, Manchester University Foundation Trust, Manchester, United Kingdom

**Keywords:** parathyroid disease, hyperparathyroidism, parathyroid gland, parathyroidectomy, calcimimetics

This Research Topic, “Recent Advances in the Treatment of Parathyroid Disease,” focuses on various aspects of treatment of primary, secondary, and tertiary hyperparathyroidism. Additionally, it discusses techniques to preserve the parathyroid glands (PTGs) during thyroidectomy. Karwacka et al. demonstrated the effect of parathyroidectomy (PTx) for primary hyperparathyroidism (PHPT) on hypertension control and left ventricular function. After PTx for PHPT, 78% of patients showed improvement in hypertension (HT). Six months post-PTx for PHPT, improvements in left atrial and ventricular function in patients with HT were demonstrated using transthoracic echocardiography. This study investigated the association between PHPT and cardiovascular disease. Li et al. analyzed the literature on cinacalcet and secondary hyperparathyroidism (SHPT). According to this meta-analysis, cinacalcet reduced the serum parathyroid hormone (PTH), calcium, phosphate, and calcium-phosphate product levels. However, cinacalcet did not improve the all-cause or cardiovascular mortality rates. Although a significant reduction in the incidence of PTx was not observed, there was a tendency towards it in cinacalcet users. Adverse events such as nausea, vomiting, and hypocalcemia were significantly more frequent in cinacalcet users. This report suggests the importance of PTx in the treatment of SHPT despite the drastic reduction in PTx numbers following the development of cinacalcet. Hiramitsu et al. reported the treatment of SHPT, focusing on PTx. They were the first to demonstrate the operative indications of PTx for SHPT and also discuss an associated preoperative imaging evaluation method. They also discussed the advantages and disadvantages of each PTx surgical procedure for SHPT and concluded that total PTx with autotransplantation and transcervical thymectomy might be the best approach. Additionally, they highlighted the importance of intraoperative neuromonitoring, intact PTH monitoring, and frozen section diagnosis. They then described the diagnosis and repeat PTx of persistent and recurrent SHPT after PTx. Moreover, they discussed medical treatment for SHPT and compared treatment outcomes between calcimimetics and PTx. Hiramitsu et al. investigated predictive factors for autograft-dependent recurrent SHPT after total PTx. In their study, multivariate analysis demonstrated that dialysis vintage and the maximum diameter of the PTG for autografts were significant contributing factors to autograft-dependent recurrent SHPT after total PTx. Receiver operating characteristic curve analysis indicated that a PTG diameter <14 mm was optimal for autografts. Additionally, they explored the correlation between pathological findings (the hyperplastic pattern of PTG used for autografts) and autograft-dependent recurrent SHPT, though they did not identify a significant relationship between the hyperplastic pattern of the PTG used for the autografts and autograft-dependent recurrent SHPT. Based on these results, they concluded that a PTG <14 mm should be used as an autograft to prevent autograft-dependent recurrent SHPT. Casella et al. investigated surgical treatment for tertiary hyperparathyroidism (THPT). They compared the total PTx with autotransplantation to the subtotal PTx for THPT. Although the study included only a small number of patients, there were no significant differences in persistent or recurrent THPT, transitory hypocalcemia, or temporary or permanent hypoparathyroidism between the two operative procedures. Statistically, significantly lower mean calcium and intact PTH levels were observed among patients with total PTx in the autotransplantation group. However, the mean calcium and intact PTH levels were within the normal ranges. These results suggest that each operative procedure for THPT should be performed following an adequate patient-informed interview, preoperative multidisciplinary discussion, and consideration of intraoperative findings. Rao et al. reported preservation of PTGs during thyroid and neck surgery. They discussed the causes of postoperative hypocalcemia, including surgical factors related to preserving the PTGs. To preserve PTGs appropriately, they detailed the anatomy of PTGs and the techniques used to identify them. They described autofluorescence using infrared cameras and carbon nanoparticles as a preservation technique, as well as outlined procedures for autotransplantation. Wang et al. investigated preservation methods for PTGs using carbon nanoparticles during endoscopic thyroid cancer surgery. Carbon nanoparticles accumulate in the lymph nodes, staining the thyroid tissue and lymph nodes black. This enables surgeons to perform a thorough lymph node dissection in the central area. Additionally, the “negative parathyroid imaging effect” of the carbon nanoparticles allows surgeons to easily identify PTGS and safely preserve parathyroid function. Mu et al. investigated the impact of recurrent laryngeal nerve (RLN) monitoring on parathyroid surgery. While the effectiveness of intraoperative neural monitoring (IONM) in thyroid surgery is well-documented, its usefulness in parathyroid surgery, especially PHPT, remains to be investigated. In the present study, the incidence of transient RLN injury was significantly lower in patients who underwent PTx for PHPT with IONM. The results of this study verified the usefulness of IONM in PTx for PHPT. Recent advancements have been reported in the treatment of parathyroid disease. We anticipate that this series of articles will contribute to the continuing refinements of surgical interventions for parathyroid disease.

## Author contributions

TH: Writing – original draft. ZM: Writing – review & editing. TA: Writing – review & editing.

